# Oxygen Displacement in Cuprates under Ionic Liquid Field-Effect Gating

**DOI:** 10.1038/srep32378

**Published:** 2016-08-31

**Authors:** Guy Dubuis, Yizhak Yacoby, Hua Zhou, Xi He, Anthony T. Bollinger, Davor Pavuna, Ron Pindak, Ivan Božović

**Affiliations:** 1 MacDiarmid Institute for Advanced Materials & Nanotechnology, Robinson Research Institute, Victoria University of Wellington, Lower Hutt 5046, New Zealand; 2 Condensed Matter Physics & Materials Science Department, Brookhaven National Laboratory, Upton, New York 11973, USA; 3 Laboratory of Physics of Complex Matter (LPMC), École Polytechnique Fédérale de Lausanne, 1015 Lausanne, Switzerland; 4 Racah Institute of Physics, Hebrew University, Jerusalem, 91904, Israel; 5 Advanced Photon Source, Argonne National Laboratory, Argonne, Illinois 60439, USA; 6 Applied Physics Department, Yale University, New Haven, Connecticut 06520, USA; 7 National Synchrotron Light Source II, Brookhaven National Laboratory, Upton, New York 11973, USA

## Abstract

We studied structural changes in a 5 unit cell thick La_1.96_Sr_0.04_CuO_4_ film, epitaxially grown on a LaSrAlO_4_ substrate with a single unit cell buffer layer, when ultra-high electric fields were induced in the film by applying a gate voltage between the film (ground) and an ionic liquid in contact with it. Measuring the diffraction intensity along the substrate-defined Bragg rods and analyzing the results using a phase retrieval method we obtained the three-dimensional electron density in the film, buffer layer, and topmost atomic layers of the substrate under different applied gate voltages. The main structural observations were: (i) there were no structural changes when the voltage was negative, holes were injected into the film making it more metallic and screening the electric field; (ii) when the voltage was positive, the film was depleted of holes becoming more insulating, the electric field extended throughout the film, the partial surface monolayer became disordered, and equatorial oxygen atoms were displaced towards the surface; (iii) the changes in surface disorder and the oxygen displacements were both reversed when a negative voltage was applied; and (iv) the c-axis lattice constant of the film did not change in spite of the displacement of equatorial oxygen atoms.

Soon after the discovery of High-Temperature Superconductivity (HTS) in cuprates^1^, it was proposed^2^ that electrostatic field could be used to control HTS at the sample surface. After years of intense research by numerous groups, it was realized that the traditional solutions used in fabrication of semiconductor field-effect transistors may not be the best approach, because of the peculiar material properties of cuprates^3^,^4^. Notably, the mobile carrier density in HTS materials is much higher than in semiconductors, typically on the scale 10^21^ to 10^22^ cm^−3^. Thus, the screening length is extremely short, just a few Ångstroms, meaning that the field-induced modulation of carrier density will largely be confined to the surface layer just one unit cell (1 UC) thick. Clearly, this imposes very stringent requirements on the HTS samples. For any conceived applications, the later should take the form of a thin film, which must be grown with an atomically smooth and perfect surface. Indeed, to observe an increase of the critical temperature (T_c_) upon electrostatic gating, the 1 UC thick HTS layer at the surface must be structurally perfect and continuous. To observe a decrease in T_c_ is in fact even more difficult, as in this case the top HTS layer should be just 1 UC thick total; if any buffer layer is placed below, it must have a lower T_c_, or not be superconducting at all. If this is not the case, the response of the buffer layer will dominate, and hide any induced change in the top HTS layer. This calls for sample engineering at an atomic-layer level that can be achieved by Atomic-Layer-by-Layer Molecular Beam Epitaxy (ALL-MBE)^5^, which provides HTS films with atomically sharp surfaces and interfaces^6^,^7^,^8^.

Nevertheless, even this is not sufficient, since the field that can be achieved with the strongest dielectrics reaches only 10^7^ V cm^−1^, one order of magnitude too low to achieve a major change in T_c_. This motivated experiments with electrolyte gating, using conducting polymers and ionic liquids, which indeed sustain much higher surface charge levels than the traditional solid dielectrics, and are therefore extensively used in ultra-capacitors.

Electrolyte gating of HTS cuprates was pioneered in the nineties by McDevitt and coworkers^9^,^10^,^11^. After being dormant for over a decade, the field has experienced a great renaissance in recent years, and apart from cuprates^8^,^12^ it has been applied to various other complex oxides^13^,^14^,^15^,^16^ as well as other correlated electron materials^17^,^18^,^19^,^20^,^21^. Changes in the free carrier concentrations have been observed^22^ as large as 8 × 10^14^ cm^−2^ - an order of magnitude more than what is obtainable using solid dielectrics. Some of the key accomplishments include illuminating studies of the quantum critical phenomena in the vicinity of the superconductor-to-insulator phase transition^8^,^12^,^23^,^24^ as well as inducing superconductivity in otherwise non-superconducting materials^25^.

However, what exactly happens inside the material subject to electrolyte gating has been subject of a vivid scientific debate. Iwasa and coworkers claimed that the effect is purely electronic, namely accumulation or depletion of electrons in the ‘channel’ material. In contrast, Parkin and coworkers claimed that, at least in VO_2_, electrolyte gating triggers oxygen electromigration, including field-induced creation of oxygen vacancies^14^. For this they presented substantial evidence including careful studies of the effect of atmosphere (oxygen vs. vacuum), as well as O^18^-O^16^ isotope substitution. Nevertheless, Okuyama *et al*. disputed this, rather ascribing the observations to a deformation of the crystal lattice of VO_2_ throughout the entire film volume^26^. This scientific disagreement shows that much still needs to be understood about the effects of electrolyte gating on complex oxides.

In this paper, we present evidence for yet another mechanism, separate from the ones cited above. Using a synchrotron-based phase-retrieval X-ray diffraction technique, we measured structural changes in a prototype cuprate, La_2−x_Sr_x_CuO_4_ (LSCO), when it is exposed to a high electric field achieved by electrolyte gating. We observe a deformation of the crystal structure that consists of significant and reversible displacements of the oxygen ions within the CuO_2_ planes in the film, but without a significant change in the lattice constant.

## Results

The specific LSCO film studied in this experiment was an ALL-MBE grown film with its active part comprised of a 5 UC thick layer of La_1.96_Sr_0.04_CuO_4_, heavily underdoped so that it was neither metallic nor superconducting. Under this layer and next to the LaSrAlO_4_ (LSAO) substrate we deposited a 1 UC thick layer of La_1.60_Sr_0.40_CuO_4_ that serves as a buffer between the active layer and the substrate. This buffer layer is necessary for structural reasons; it facilitates synthesis of a nearly perfect single-crystal underdoped LSCO film. While the bulk samples, including thicker films, of such highly overdoped LSCO are metallic, in this particular configuration this buffer remains insulating, likely due to charge leakage and localization, as we have verified by transport measurements on this film and hundreds of similar heterostructures.

**Figure 1:**
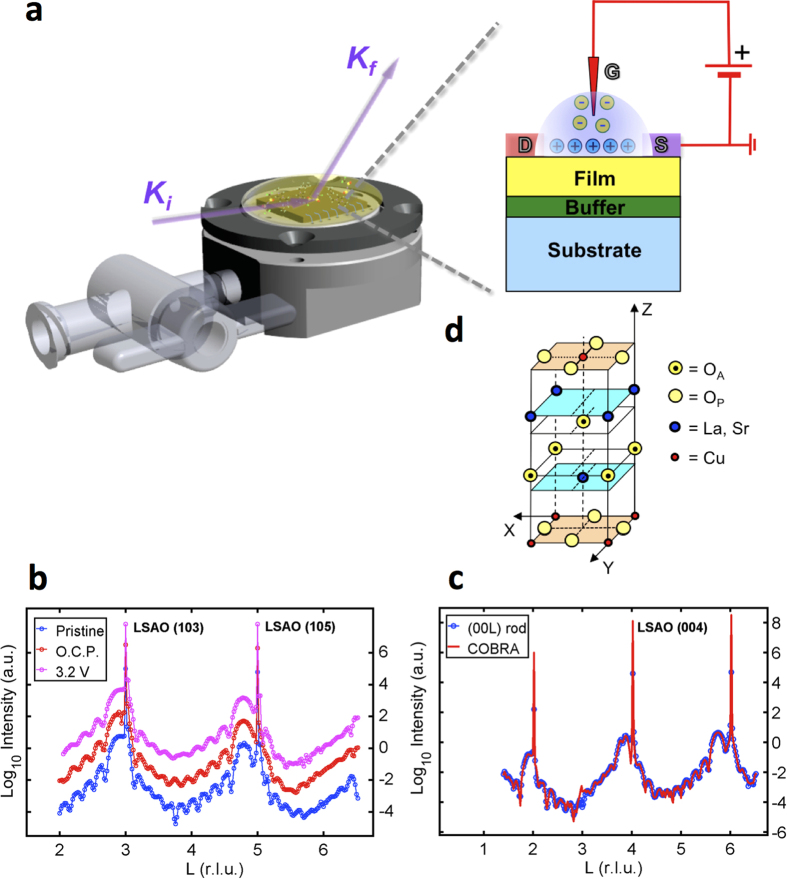
*In-situ* structural investigations of electrolyte-gated La_1.96_Sr_0.04_CuO_4_ thin film epitaxially grown on a LaSrAlO_4_ (LSAO) substrate. (**a**) Schematics of *in-situ* surface X-ray diffraction measurements and ionic-liquid gating experimental setup. *K*_*i*_ and *K*_*f*_ are incident and diffracted beam vectors, respectively. The bias voltage is applied between a contact wire bonded to the sample surface (ground) and Pt wire (gate) during ionic liquid gating operation. (**b**) The diffraction intensity along the (1 0 L) non-specular Bragg rod with the variation of bias voltage conditions. Blue: pristine sample; red: under open-circuit condition; magenta: under V_g_ = 3.2 V bias. The curves have been displaced for clarity. The reciprocal lattice unit (r.l.u.) in the film = 4.78 nm^−1^. (**c**) Blue open circles with line: the diffraction intensity measured along a representative specular Bragg rod of the La_1.96_Sr_0.04_CuO_4_ thin film epitaxially grown on a LaSrAlO_4_ substrate. Red solid line the diffraction intensity calculated from the COBRA-determined electron density. (**d**) Half crystallographic unit cell with the equatorial oxygen atoms in the CuO_2_ plane labelled O_P_ and the apical oxygens labelled O_A_.

Using standard photolithographic techniques and a special wet chemical sample cell (depicted schematically in Fig. 1a and described in detail in the Methods Section), the LSCO thin film was prepared for application of electrolyte gating using the Ionic Liquid (IL) 1-ethyl-3-methylimidazolium dicyanamide.

The X-ray diffraction intensities were measured under 4 different conditions. (a) pristine sample; (b) sample surface in contact with the IL, open circuit potential between the film and an electrode immersed in the IL; (c) a gate voltage V_g_ = 3.2 V applied between the film and the electrode; and (d) V_g_ = −2.5 V applied between the film and the electrode. We measured a full set of X-ray diffraction intensities along 6 symmetry inequivalent Bragg rods under each one of the above conditions. The sample was allowed to stabilize for approximately 1 hour after each change of gate voltage before starting the measurement.

In Fig. 1b we show the diffraction intensity along the (1 0 L) Bragg rod measured under conditions a, b and c. The diffraction intensity curves differ significantly at different measuring conditions. Notice that just putting the sample in contact with the IL changes the diffraction curve significantly.

The diffraction intensity along the substrate defined Bragg rods contains detailed structural information about the system atomic structure. Specifically it contains the information about the system folded structure electron density. The folded structure is obtained by translating each atom laterally into one substrate defined two-dimensional (2D) unit cell using substrate defined 2D unit cell vectors^27^. However, this information cannot be obtained directly from the diffraction data because the electron density at any point in real space affects the diffraction intensity in the entire reciprocal space. So to obtain the real space electron density we have analyzed the data using the Coherent Bragg Rod Analysis (COBRA) method^27^. The method is described in detail in references^27^,^28^,^29^ and was previously used to determine with sub-Ångstrom resolution the complete atomic structure of epitaxial LSCO ultrathin films^28^,^29^. In the present case we used the nominal structure as our reference structure; the diffraction phases were determined using the COBRA approximation followed by the ‘difference map’ method^30^ yielding the Complex Scattering Factors (CSF’s). Fourier transforming the CSF’s into real space resulted in the 3D electron density. We compared the experimental diffraction intensities with the diffraction intensities calculated using the electron densities determined by COBRA. An example of the comparison between calculated and measured intensities for the (0 0 L) Bragg rod for the pristine sample is shown in Fig. 1c. A comparison for the complete set of 6 Bragg rods for the pristine sample is provided in Supplementary Information Figs S1 and S2. Similar fit quality was obtained for the sets of 6 Bragg rods for the different sample conditions studied.

**Figure 2:**
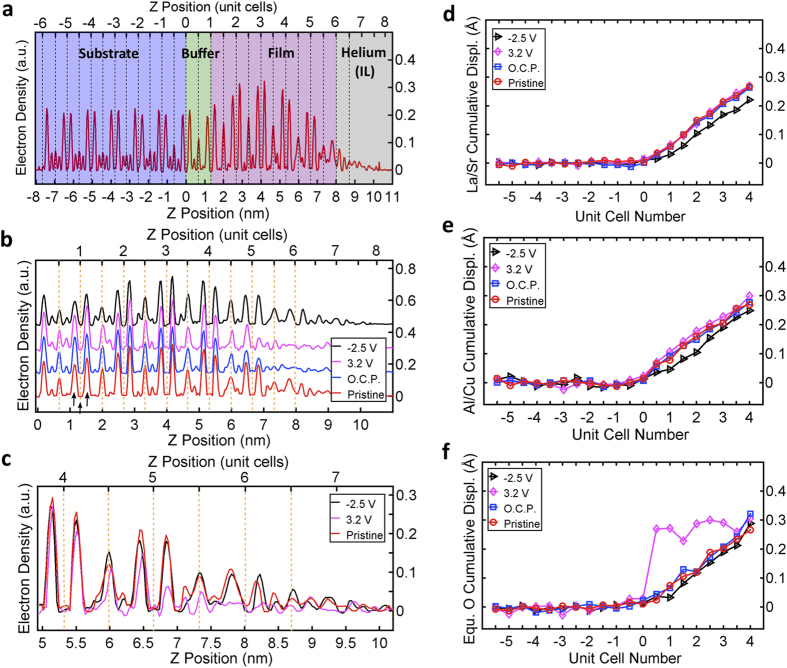
The electron density profiles and atomic displacements determined by COBRA, for various bias voltage conditions. In the figure legends, O.C.P. = open circuit condition. (**a**) The electron density profiles and atomic displacements determined by COBRA for the pristine sample along the [0 0 Z] atomic columns of the unit cell. The electron densities are graphed as a function of Z (nm) and Z (unit cells). Z = 0 nm (unit cells) is the position of the geometric interface between the substrate and the 1 UC buffer. For the substrate, the conversion is 1 unit cell = 1.262 nm, while for the film, we used the average film lattice spacing 1 unit cell = 1.315 nm. The substrate, buffer, film, and helium regions are indicated for the pristine sample, for other cases helium is replaced by the IL. Note that the last unit cell before the film-helium interface is incomplete or exhibits only partial registry with the substrate. (**b**) An expanded view within the buffer, film, and helium region. Notice the pronounced difference between the data for V_g_ = 3.2 V and V_g_ = −2.5 V bias voltage conditions, and the similarity between the data for open circuit and V_g_ = 3.2 V conditions in the region of Z > 6 nm. (**c**) An expanded view of the electron density profile in the region of 5 nm < Z < 10 nm. The average cumulative displacements (**d**) of La/Sr atoms above and below Cu, (**e**) of the Cu atoms, and (**f** ) of the equatorial oxygen (O_P_) atoms measured for the atoms in each half unit cell with zero indicating the substrate/buffer interface. All cumulative atomic displacements are relative to a reference grid composed of the La/Sr and Al substrate atoms and extended throughout the entire sample. The slope in the displacement curves indicates that the size of unit cells above the substrate/film interface is larger than that of the substrate by 0.065 nm/unit cell on average.

The electron density along the [0 0 Z] line perpendicular to the surface going through La/Sr, O_A_, and Cu atoms is shown in Fig. 2a. The peaks at Z < 0 belong to the LaSrAlO_4_ substrate. At Z > 0 Cu replaces the Al and at Z > 6 nm the peaks become smaller indicating that the atomic layers no longer cover the entire surface. Inspection of the electron densities along the [0 0 Z] lines obtained under different conditions in Fig. 2b show that they do not change at Z < 6 nm. On the other hand they do change at Z > 6 nm as shown in Fig. 2c.

Notice that the peaks corresponding to the sample in contact with the IL and under 3.2 V are smaller than those of the pristine sample. The application of V_g_ = −2.5 V reverses the situation and the electron densities of the pristine and V_g_ = −2.5 V are almost equal. The fact that the peaks diminish means that the atoms in the film in contact with the IL are no longer accurately registered relative to the substrate. However the application of V_g_ = −2.5 V restores this registry.

The voltage applied between the film and the IL affects the structure in yet another way. In Fig. 2d we present the displacement of the La/Sr atoms relative to the positions of corresponding atoms in the reference frame defined as the substrate lattice extended throughout the entire sample. The displacements of the Al/Cu atoms are shown in Fig. 2e. In the substrate (unit cell number = −5 to 0) the displacements of the Al atoms are of course zero. At unit cell number = 1 and higher, the displacements increase linearly, meaning that the Z-axis lattice constant of the film (Z_0_) is larger than that of the substrate. This remains true for the sample under all four different conditions. In Fig. 2f we also show the displacements of equatorial oxygen atoms (O_P_), i.e., those that belong to the ‘CuO_2_ planes’. Notice that when V_g_ = 3.2 V is applied the displacements are positive, namely the oxygen atoms are displaced towards the surface. The oxygen atoms are restored to their original positions when V_g_ = −2.5 V is applied. Since the equatorial oxygen displacements are a key result, we show the electron density curves from which these displacements were derived in Supplementary Information Figs S3 and S4 for the O.C.P. and 3.2 V cases respectively.

**Figure 3:**
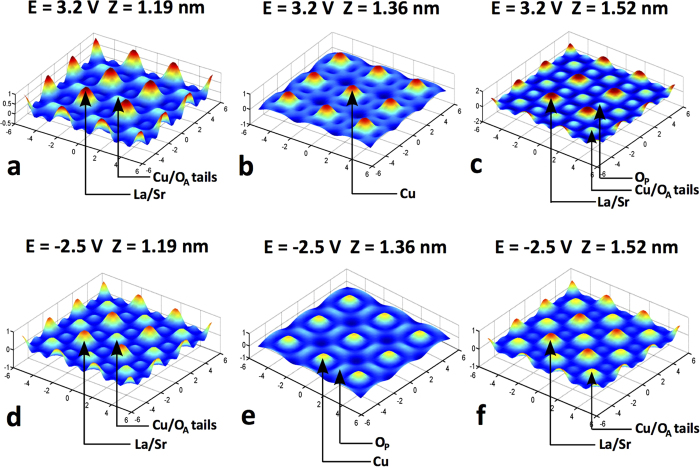
2D electron density maps in the atomic planes parallel to the surface of the sample at two extreme bias voltages: (**a–c**) under V_g_ = 3.2 V bias voltage at Z = 1.19, 1.36, 1.52 nm, respectively; (**d–f**) under V_g_ = −2.5 V bias voltage at Z = 1.19, 1.36, 1.52 nm, respectively. The Z positions for the 2D electron density maps are marked with arrows in Fig. 2b. The atoms contributing electron density to the different peaks are indicated. O_p_ designates CuO_2_ plane oxygens; the peaks marked Cu/O_A_ tails are due to the electron density tails of the copper ion on one side and the apical oxygen ion on the other.

In Fig. 3a–c we show the electron density in three planes parallel to the surface under V_g_ = 3.2 V bias voltage. The plane at Z = 1.36 nm goes through the Cu atoms at Z = 1 UC while the planes at Z = 1.19 and Z = 1.52 nm go through the La/Sr atoms below and above the Cu plane, respectively. Figure 3d–f show the electron densities in the same planes but under V_g_ = −2.5 V. Notice that, under V_g_ = 3.2 V, the equatorial oxygen (O_P_) atoms are missing from the plane at Z = 1.36 nm (Fig. 3b), while they show up in the plane at Z = 1.52 nm (Fig. 3c) but not in the plane at Z = 1.19 nm (Fig. 3a) indicating that they moved towards the surface. After applying V_g_ = −2.5 V the oxygen atoms show up again in the plane at Z = 1.36 nm (Fig. 3e) namely in the Cu plane and are barely seen on the planes at Z = 1.19 nm (Fig. 3d) and Z = 1.52 nm (Fig. 3f) indicating they moved back to the Cu plane. The equatorial oxygen atoms are again displaced when V_g_ = 3.2 V is applied and the circuit is then opened.

## Discussion

An insight into this behavior can be obtained by calculating the electric field and the energy difference between the Fermi level and the top of the valence band as a function of the distance from the substrate. This was accomplished by using the following model. The Sr ions are treated as acceptors with acceptor energy *E*_*a*_ relative to the top of the valence band and concentration *N*_*a*_. The charge density as a function of position *z* is given by:


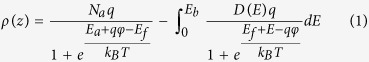


Here, *q* is the electron charge, *φ* is the electrical potential, *D(E)* is the density of states, *E* is the absolute value of the energy measured relative to the top of the valence band, and *E*_*f*_ is the Fermi energy. Notice that *E*_*f*_ *−* *qφ* is the Fermi energy relative to the top of valence band. The electrical potential and electric field satisfy the electrostatic equations:


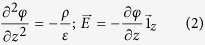


**Figure 4:**
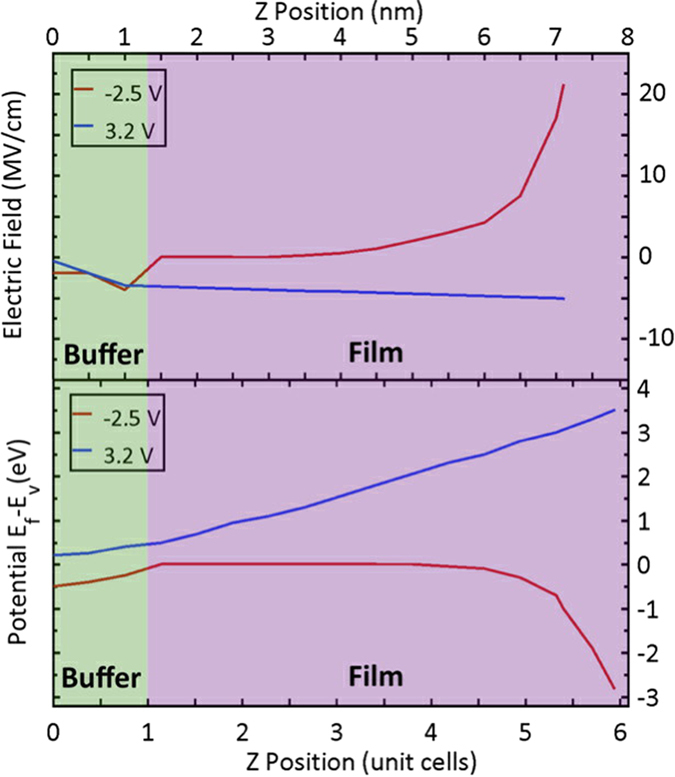
(**a**) The electric field as a function of distance from the substrate. (**b**) The difference between the Fermi energy *E*_*f*_ and the top of valence band energy *E*_*v*_ as a function of distance from the substrate. For both plots, the red curve is for an applied voltage of V_g_ = −2.5 V, the blue curve is for V_g_ = 3.2 V. The buffer and film regions are indicated.

In the present case Sr is expected to be always Sr^2+^ therefore *E*_*a*_ < −10 eV. The Sr concentration in the metal film is 0.4 per unit cell and in the semiconductor it is 0.04 per unit cell. The density of states is taken from ref. 31 Figure 4c inset and the dielectric constant is taken^32^ as *ε* = 30.

The resulting electric field and the energy difference between the Fermi level and the top of the valence band are shown in Fig. 4a,b, respectively. The red and blue curves correspond to an applied voltage of V_g_ = −2.5 V and V_g_ = 3.2 V, respectively. Notice that when the positive voltage is applied the film is partially depleted of holes, which makes it more insulating so that the electric field can penetrate the film. In contrast, when the applied voltage is negative, holes are injected into the film making it more metallic, and consequently the electric field is screened within a very short distance.

Comparing the equatorial oxygen displacements with the electric fields calculated above indicates that the oxygen displacements are not a linear response to the electric field. Close to the surface the electric field is the largest while the displacements in the top two unit cells are negligibly small. This behavior suggests that the force opposing the displacement decreases with the distance from the surface.

One important question concerning this and other experiments with electrolyte gating of complex oxides is what exactly happens to the gated film. As discussed earlier, two prominent candidate explanations have been put forward. Iwasa and coworkers argued in favor of a purely electronic mechanism, where the primary effect is electron accumulation (for positive gate voltage) or depletion (for negative gate voltage) near the surface. However, Parkin and coworkers have shown that at least in VO_2_, electrolyte gating results in massive electromigration of oxygen and changes in the concentration of oxygen vacancies. One way to differentiate between the two is by X-ray diffraction. Electrochemical processes such as ion intercalation or extraction generally lead to observable changes in the crystal structure and lattice constants. In contrast, given the very short screening length in metals, for a purely electronic mechanism any changes should be negligibly small restricted to a very thin layer (less than 1 nm thick) near the surface. Since X-ray diffraction probes the entire film thickness, one would then expect no changes in the lattice constants.

It is well known that undoped LCO and underdoped LSCO are quite open to oxygen intercalation in the interstitial position in-between two neighboring La-O layers, even without an applied electric field^33^. For example, LCO can be driven metallic and superconducting by low-temperature annealing in ozone, and back to the insulating state by annealing in vacuum. These processes affect notably the crystal lattice parameters, while the magnitude of the effect depends on the film thickness. The effect is quite dramatic in very thin films; e.g., in a 10 UC (d = 13 nm) thick film, the *c*-axis lattice constant was found^33^ to vary by as much as 0.3 Å, i.e., by more than 2%.

However, in the present experiments we have seen no changes in the unit cell size, for either polarity of the gate voltage, to the experimental uncertainty of ± 0.002 Å. This is two orders-of-magnitude lower than the maximal effect we would expect if the film were fully charged by oxygen intercalation. In our opinion, this rules out oxygen electrochemistry as the dominant mechanism of electrolyte gating of LSCO.

In conclusion, we have studied the structural changes induced in a 5 UC La_1.96_Sr_0.04_CuO_4_ film epitaxially grown on a LaSrAlO_4_ substrate with a 1 UC La_1.60_Sr_0.40_CuO_4_ buffer layer. Ultra-high electric fields were induced in the film by applying a gate voltage between the film and an ionic liquid in contact with it. Measuring the diffraction intensity along the substrate-defined Bragg rods and analyzing the results using the COBRA method we obtained the 3D electron density in the film, the buffer layer, and the topmost atomic layers of the substrate. The diffraction intensities and the electron densities were determined under 4 conditions: (a) pristine sample; (b) sample exposed to IL open circuit; (c) V_g_ = 3.2 V; and (d) V_g_ = −2.5 V. The main structural observations were as follows.

- No structural changes are observed even in atomic monolayers that do not fully cover the surface when the voltage is negative, holes are injected into the film making it more metallic and screening the electric field.
- When the voltage is positive, the film is depleted of holes thus becoming more insulating, the electric field extends throughout the film, the monolayer that only partially covers the surface becomes disordered, and planar oxygen atoms are displaced towards the sample surface.
- The changes in surface disorder and the oxygen displacements are both reversed when V_g_ = −2.5 V is applied.
- To within our experimental accuracy, the c-axis lattice constant of the film does not change in spite of the displacement of planar oxygen atoms, ruling out oxygen electrochemistry as the dominant mechanism of electrolyte gating of LSCO.

The results presented here show that, by applying ultrahigh electric fields using ionic liquid gating, significant structural changes are induced in LSCO films. These distortions can be quantitatively measured by surface x-ray diffraction combined with the COBRA phase retrieval method.

## Methods

### Sample preparation and design of the wet chemical cell for X-ray scattering

A thin layer (10 nm) of gold was deposited on top of LSCO film *in-situ* immediately after growth. This cap layer protects the film surface during photolithographic processing. Six other films of identical or very similar composition were also synthesized for comparison. The one for which the data are shown appeared the best from the viewpoint of synthesis and characterization prior to the X-ray experiments.

Using standard photolithography, we patterned the films into devices allowing for 4-point-contact transport measurements while simultaneously applying the gate voltage from an additional separate gate electrode. Thick gold layers were subsequently deposited on the contact pads, and then the thin gold cap layer deposited *in-situ* was removed by chemical etching from the active part of the device, exposing a clean La_1.96_Sr_0.04_CuO_4_ film surface to the IL. For the IL, we chose 1-ethyl-3-methylimidazolium dicyanamide, because we verified that its properties are not affected by X-ray radiation at the dose levels as used in the present experiment.

In order to apply electrolyte gating to a sample exposed to a synchrotron X-ray beam as used for COBRA, we developed a dedicated wet chemical cell^34^. The cell is illustrated schematically in Fig. 1a. It was designed to accommodate a 10 × 10 × 1 mm^3^ sample, and made out of Kel-F polymer. To hold the sample and seal the electrical feedthroughs we used TorrSeal^®^ High Vacuum Epoxy. To close the cell, it was covered by a Kapton film. These materials were chosen for their high chemical inertia and vacuum compatibility. The cell size was kept at the minimum, and the shape designed to allow for a grazing angle access to the film surface from all directions, except for a small shadow from the wire-bonded electrical connections, which were made as shallow as possible. The contacts were covered with epoxy, so that only the active device surface was exposed to the IL. Multiple electrical connections to the sample allowed for charging as well as for an *in-situ* measurement of the electrical resistance, in the 4-point contact configuration. The film was patterned in such a way as to avoid any parallel conductance path through the portions of the film that were not exposed to the IL. Moreover, we took precautions to eliminate any effects of the interaction between the epoxy and film; the design makes sure that the 4-point contact resistance is only measured in the clean film. The resistance was measured only intermittently after changing gate voltage, when we could access the sample. This was used to ensure that a stable state was reached before starting the X-ray measurement. Under 3.2 V the resistance increased above 17 MOhm, while with −2.5 V the resistance dropped to 3.3 kOhm after it was left to charge for 1 hour. These results agree with more detailed measurements of the resistance on a similar sample reported elsewhere^8^.

A thin platinum wire, connected to the IL volume using a separate feedthrough, served as the gating electrode. It was wound around the sample in order to provide for a large gate surface all around the sample, and thus ensure uniform charging of the whole sample surface exposed to IL, about 80 mm^2^.

A sheet of Kapton (7 μm thick) was stretched over the sample, serving as an X-ray window. To further reduce the thickness of the IL layer covering the sample, the cell was kept under a slight negative pressure. For this reason, a syringe with a valve was connected to the cell. Before the start of the experiment, a moderate vacuum was pulled on the cell using the syringe, and the valve was then closed. Visual inspection of the sample allows us to easily determine whether the cell is vacuum-tight. Typical sources of leaks are pinholes in the Kapton film, occurring at the places where Kapton is pressed against sharp edges of the sample. In order to alleviate this problem, we slightly blunted the sample’s edges. In order to protect the Kapton film during exposure to X-rays, it was kept in an inert atmosphere. For this reason, the cell was mounted in a custom vacuum chamber closed by a Beryllium dome and flooded with Helium. Note that the closed cell cannot be kept under vacuum. There must at all times be a significant pressure difference between the inside of the cell, and its environment. If the outside pressure reached a high vacuum the Kapton film would be delaminated from the sample surface, which would result in a non-uniform IL film on the sample.

### Surface X-ray scattering

The diffraction intensities were measured at ID33 beamline at the Advanced Photon Source, Argonne National Laboratory, Argonne IL. The measuring system consisted of a six-circle Kappa-type goniometer and Pilatus 100 K area detector.

## Additional Information

**How to cite this article**: Dubuis, G. *et al*. Oxygen Displacement in Cuprates under Ionic Liquid Field-Effect Gating. *Sci. Rep.* **6**, 32378; doi: 10.1038/srep32378 (2016).

## Supplementary Material

Supplementary Information

## References

[b1] Bednorz J. G. & Müller K. A. Possible high T_c_ superconductivity in the Ba-La-Cu-O system. Z. Phys. B Con. Mat. 64, 189 (1986).

[b2] Brazovskii S. A. & Yakovenko V. M. Possible superconductivity on the junction surface of dielectric La_2_CuO_4_. Phys. Lett. A 132, 290 (1988).

[b3] Ahn C. H., Triscone J.-M. & Mannhart J. Electric field effect in correlated oxide systems. Nature 424, 1015 (2003). 12944958 10.1038/nature01878

[b4] Ahn C. H. . Electrostatic modification of novel materials. Rev. Mod. Phys. 78, 1185 (2006).

[b5] Božović I. Atomic-layer engineering of superconducting oxides: yesterday, today, tomorrow. IEEE Trans. Appl. Supercond. 11, 2686 (2001).

[b6] Gozar A. M. . Interface superconductivity between a metal and a Mott insulator. Nature 455, 782 (2008). 18843365 10.1038/nature07293

[b7] Logvenov G. Y., Gozar A. M. & Božović I. High-temperature superconductivity in a single copper-oxygen plane. Science 326, 699 (2009). 19900926 10.1126/science.1178863

[b8] Bollinger A. T. . Superconductor–insulator transition in La_2−x_Sr_x_CuO_4_ at the pair quantum resistance. Nature 472, 458 (2011). 21525929 10.1038/nature09998

[b9] Haupt S. G., Riley D. R. & McDevitt J. T. Conductive polymer/high-temperature superconductor composite structures. Adv. Mater. 5, 755 (1993).

[b10] Haupt S. G. . Reversible modulation of superconductivity in YBa_2_Cu_3_O_7−δ_/polypyrrole sandwich structures. Proc. SPIE 2158, 238 (1994).

[b11] Clevenger M. B., Jones C. E., Haupt S. G., Zhao J. & McDevitt J. T. Organic conductor/high-Tc superconductor bilayer structures. Proc. SPIE 2697, 508 (1996).

[b12] Leng X., Garcia-Barriocanal J., Bose S., Lee Y. & Goldman A. M. Electrostatic control of the evolution from a superconducting phase to an insulating phase in ultrathin YBa_2_Cu_3_O_7−x_ films. Phys. Rev. Lett. 107, 027001 (2011). 21797633 10.1103/PhysRevLett.107.027001

[b13] Ueno K. . Electric-field-induced superconductivity in an insulator. Nat. Mater. 7, 855 (2008). 18849974 10.1038/nmat2298

[b14] Jeong J. . Suppression of metal-insulator transition in VO_2_ by electric field–induced oxygen vacancy formation. Science 339, 1402 (2013). 23520104 10.1126/science.1230512

[b15] Nojima T. . Hole reduction and electron accumulation in YBa_2_Cu_3_O_y_ thin films using an electrochemical technique: Evidence for an n-type metallic state. Phys. Rev. B 84, 020502 (2011).

[b16] Nakano M. . Collective bulk carrier delocalization driven by electrostatic surface charge accumulation. Nature 487, 459 (2012). 22837001 10.1038/nature11296

[b17] Ye J., Zhang Y. J., Kasahara Y. & Iwasa Y. Interface transport properties in ion-gated nano-sheets. Eur. Phys. J. Spec. Top. 222, 1185 (2013).

[b18] Ye J. . Gate induced superconductivity in layered material based electronic double layer field effect transistors. Physica C 470, S682 (2009).

[b19] Kasahara Y. . Electric-field-induced superconductivity detected by magnetization measurements of an electric-double-layer capacitor. J. Phys. Soc. Jpn. 80, 023708 (2011).

[b20] Ye J. . Liquid-gated interface superconductivity on an atomically flat film. Nat. Mater. 9, 125 (2009). 19935665 10.1038/nmat2587

[b21] Ye J. . Gate-induced superconductivity in layered-material-based electric double layer transistors. Chinese Phys. C 400, 022139 (2012).

[b22] Yuan H. . Electrostatic and electrochemical nature of liquid-gated electric-double-layer transistors based on oxide semiconductors. J. Am. Chem. Soc. 132, 18402 (2010). 21141862 10.1021/ja108912x

[b23] Ueno K. . Effective thickness of two-dimensional superconductivity in a tunable triangular quantum well of SrTiO_3_. Phys. Rev. B 89, 020508 (2014).

[b24] Zeng S. W. . Two-dimensional superconductor-insulator quantum phase transitions in an electron-doped cuprate. Phys. Rev. B 92, 020503 (2015).

[b25] Ueno K. . Discovery of superconductivity in KTaO_3_ by electrostatic carrier doping. Nat. Nanotechnol. 6, 408 (2011). 21602813 10.1038/nnano.2011.78

[b26] Okuyama D. . Gate-tunable gigantic lattice deformation in VO_2_. Appl. Phys. Lett. 104, 023507 (2014).

[b27] Sowwan M. . Direct atomic structure determination of epitaxially grown films: GaAs: Gd_2_O_3_. Phys. Rev. B 66, 205311 (2002).

[b28] Zhou H. . Anomalous expansion of the copper-apical oxygen distance in superconducting La_2_CuO_4_ - La_1.55_Sr_0.45_CuO_4_ bilayers. Proc. Natl. Acad. Sci USA 107, 8103 (2010). 20404212 10.1073/pnas.0914702107 PMC2889550

[b29] Yacoby Y., Zhou H. & Pindak R. & Božović, I. Atomic-layer synthesis and imaging uncover broken inversion symmetry in La_2−x_Sr_x_CuO_4_ films. Phys. Rev. B 87, 014108 (2013).

[b30] Elser V. Solution of the crystallographic phase problem by iterated projections. Acta Cryst. 59, 201 (2003). 10.1107/s0108767303002812 12714770

[b31] Wu J. . Anomalous independence of interface superconductivity on carrier density. Nat. Mat. 12, 877 (2013). 10.1038/nmat3719 23913171

[b32] Chen C. Y., Birgeneau R. J., Kastner M. A., Preyer N. W. & Thio T. Frequency and magnetic-field dependence of the dielectric constant and conductivity of La_2_CuO_4+y_. Phys. Rev. B 43, 392 (1991). 10.1103/physrevb.43.392 9996223

[b33] Bozovic I., Logvenov G., Belca I., Narimbetov B. & Sveklo I. Epitaxial Strain and Superconductivity in La_2−x_Sr_x_CuO_4_ Thin Films. Phys. Rev. Lett 89, 107001–107004 (2002). 12225215 10.1103/PhysRevLett.89.107001

[b34] Dubuis G. PhD thesis, *École Polytechnique Fédérale de Lausanne (Switzerland*) 2014.

