# Supplementary material for: Oxygen Displacement in Cuprates under Ionic Liquid Field-Effect Gating
Source: Sci Rep. 2016 Aug 31;6:32378. doi: 10.1038/srep32378 (PMC5006154; doi:10.1038/srep32378)
Supplement: Supplementary Information [file srep32378-s1.pdf]

Supplementary Information

Oxygen Displacement in Cuprates under Ionic Liquid Field-Effect Gating

Guy Dubuis, Yizhak Yacoby, Hua Zhou, Xi He, Anthony T. Bollinger, Davor Pavuna, Ron Pindak and *Ivan I. Božović*

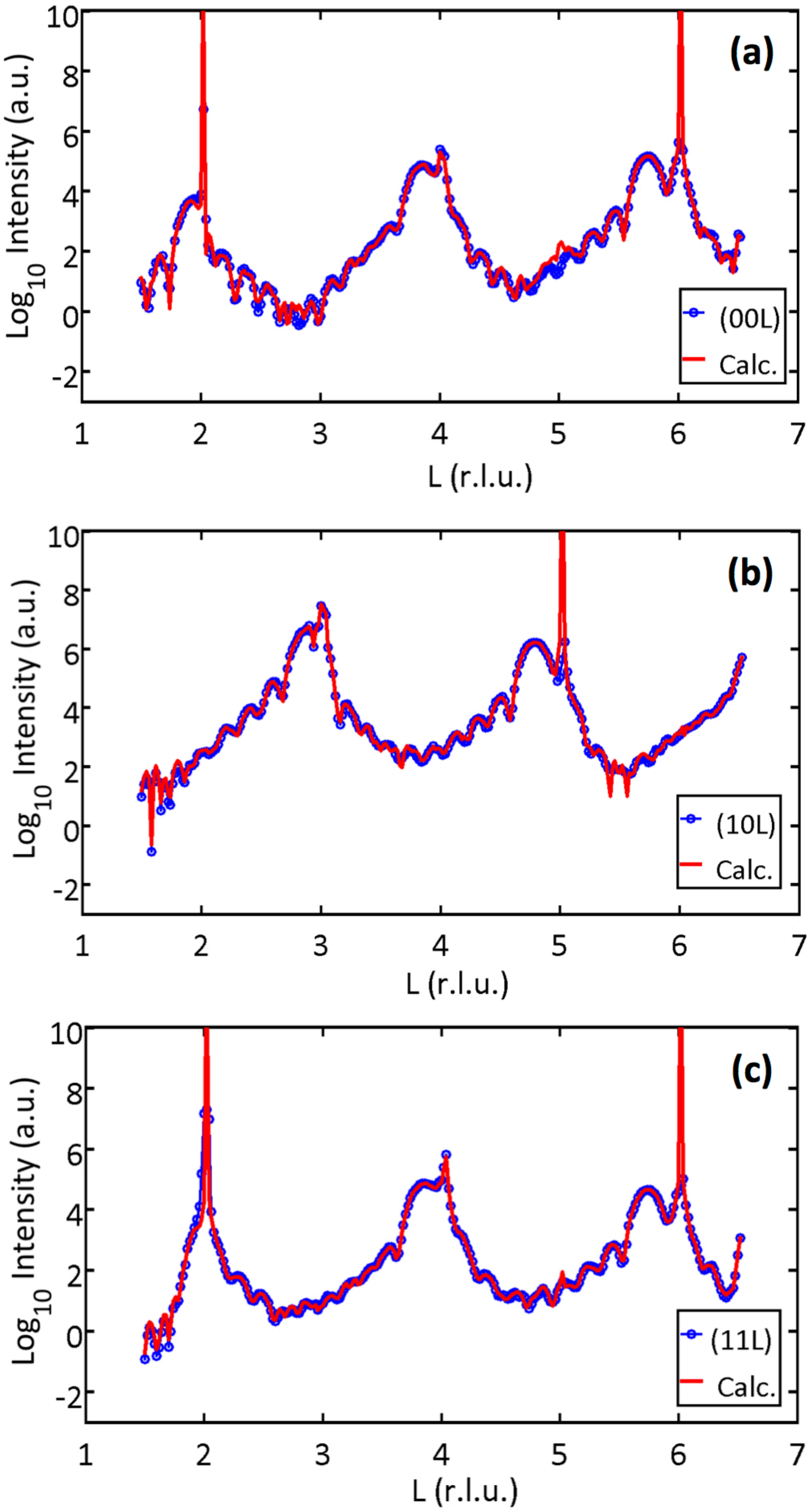
(a)
Log10 Intensity (a.u.)
L (r.l.u.)
(00L)
Calc.
(b)
Log10 Intensity (a.u.)
L (r.l.u.)
(10L)
Calc.
(c)
Log10 Intensity (a.u.)
L (r.l.u.)
(11L)
Calc.

Supplementary Figure S1. The calculated and measured diffraction intensities for a 5 UC $La_{1.96}Sr_{0.04}CuO_4$ thin film epitaxially grown on a $LaSrAlO_4$ substrate with a 1 UC buffer layer of $La_{1.60}Sr_{0.40}CuO_4$. This ‘pristine’ sample was measured before addition of an ionic liquid. Blue open circles with line is the measured diffraction intensity; red solid line is the diffraction intensity calculated from the COBRA-determined electron density for (a) the (0 0 L) Bragg rod, (b) the (1 0 L) Bragg rod, and (c) the (1 1 L) Bragg rod.

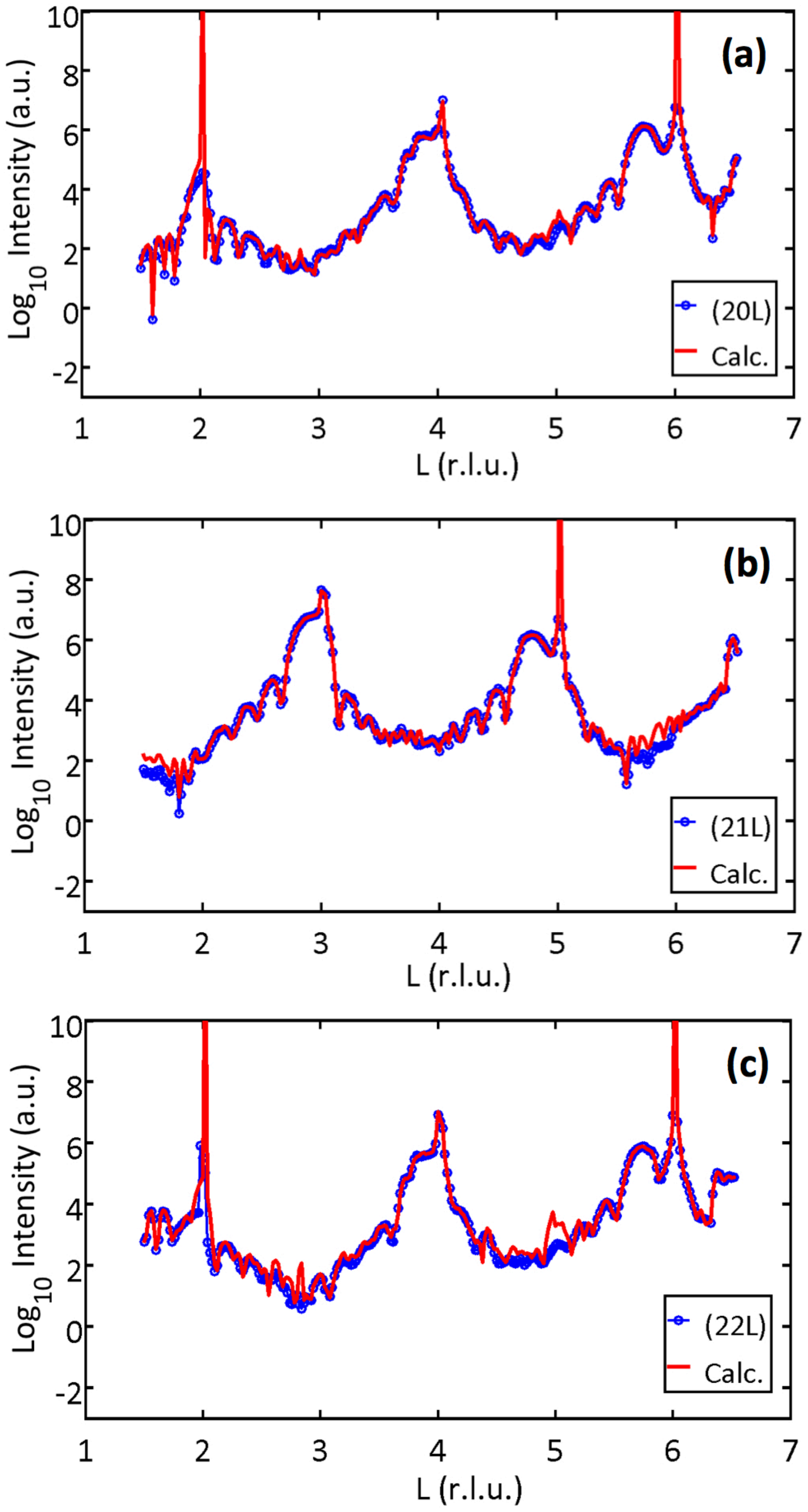

(a)
(20L)
Calc.
(b)
(21L)
Calc.
(c)
(22L)
Calc.
Log_{10} Intensity (a.u.)
L (r.l.u.)

Supplementary Figure S2. The calculated and measured diffraction intensities for the same 'pristine' sample studied in Supplementary Fig. S1. Blue open circles with line is the measured diffraction intensity; red solid line is the diffraction intensity calculated from the COBRA-determined electron density for (a) the (2 0 L) Bragg rod, (b) the (2 1 L) Bragg rod, and (c) the (2 2 L) Bragg rod. When combined with the Bragg rods in Supplementary Fig. S1, these data provide a complete set of inequivalent Bragg rods extending 7 r.l.u. normal to the substrate and 2 r.l.u. x 2 r.l.u. in-plane.

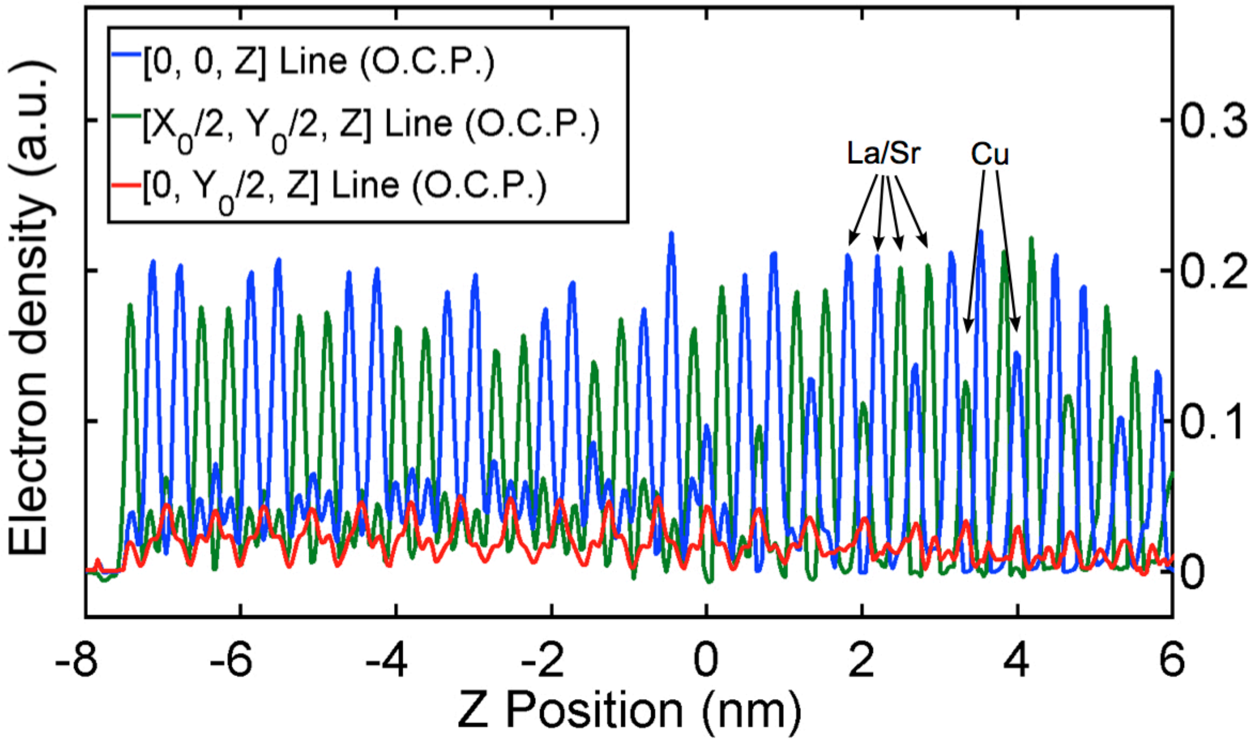

Supplementary Figure S3. The electron density of the O.C.P. sample along the [0, 0, Z] line (*blue*), the [X_0/2, Y_0/2, Z] line, (*green*), and along the [0, Y_0/2, Z] line (*red*) where X_0 and Y_0 are lattice constants along the X and Y directions respectively. Referring to the half unit cell in Fig. 1d, the [0, 0, Z] line passes through Cu-O_A-La/Sr-La/Sr-O_A atoms in each unit cell, the [X_0/2, Y_0/2, Z] line passes through La/Sr-O_A-Cu-O_A-La/Sr atoms in each unit cell, and the [0, Y_0/2, Z] line passes through two equatorial (O_P) oxygens in each unit cell. The peaks associated with a single La/Sr pair of atoms and a single Cu atom is indicated by labelled arrows along the [0, 0, Z] and [X_0/2, Y_0/2, Z] lines. In the substrate the Cu atoms are replaced by Al atoms. Note that the peaks in the electron density for O_P atoms in the red curve are centered on the peaks in the electron densities for the Cu atoms in the blue and green curves.

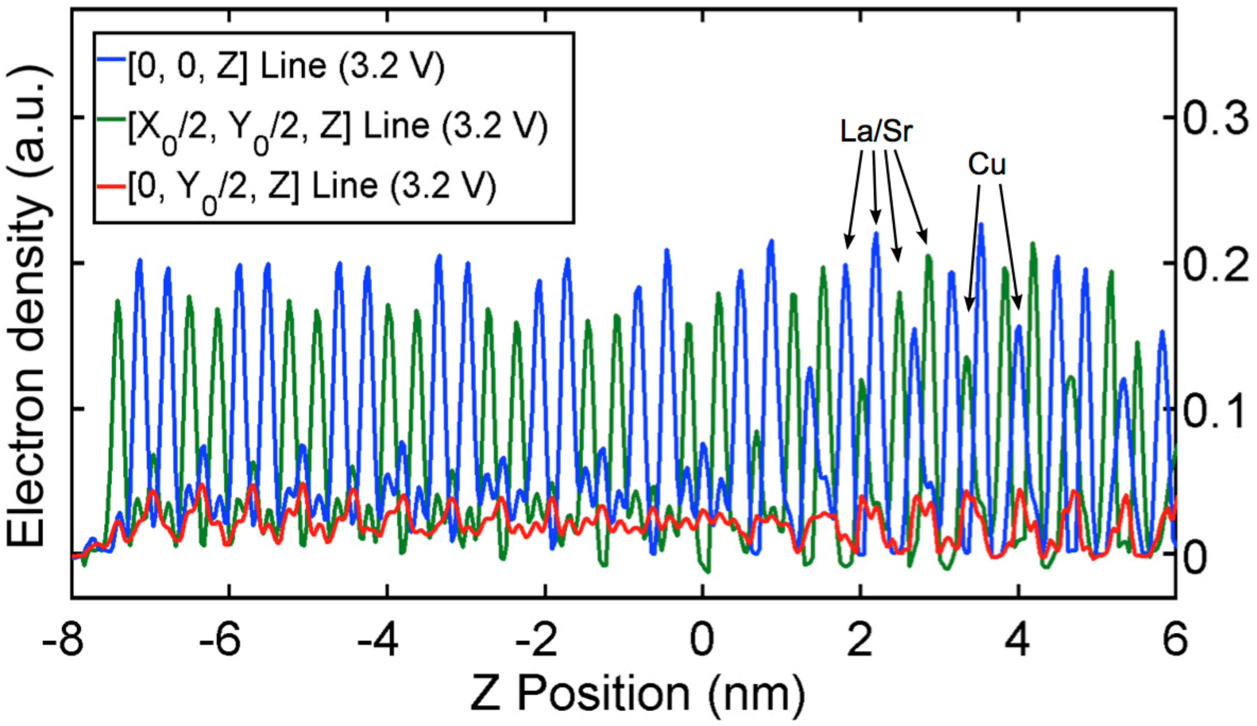

Supplementary Figure S4. Same as Supplementary Figure S3 except for the sample under 3.2 V. Notice that the peaks in the electron densities for the equatorial (O_P) oxygens peaks (*red curve*) for 0<Z<4 unit cells are displaced relative to the Cu peaks (*blue and green curves*).
